# Synchronous Wearable Wireless Body Sensor Network Composed of Autonomous Textile Nodes

**DOI:** 10.3390/s141018583

**Published:** 2014-10-09

**Authors:** Peter Vanveerdeghem, Patrick Van Torre, Christiaan Stevens, Jos Knockaert, Hendrik Rogier

**Affiliations:** 1 Department of Information Technology (INTEC), Ghent University, St. Pietersnieuwstraat 41, 9000 Ghent, Belgium; E-Mails: patrick.vantorre@ugent.be (P.V.T.); hendrik.rogier@ugent.be (H.R.); 2 Department of Industrial System and Product Design (ISP), Ghent University, Graaf Karel de Goedelaan 5, 8500 Kortrijk, Belgium; E-Mails: christiaan.stevens@ugent.be (C.S.); Jos.Knockaert@UGent.be (J.K.); 3 Department of Industrial Technology and Construction (IT&C), Ghent University, Valentin Vaerwyckweg 1, 9000 Ghent, Belgium

**Keywords:** wireless sensors, body-centric, diversity, ISM band, textile antennas, situational awareness, event detection

## Abstract

A novel, fully-autonomous, wearable, wireless sensor network is presented, where each flexible textile node performs cooperative synchronous acquisition and distributed event detection. Computationally efficient situational-awareness algorithms are implemented on the low-power microcontroller present on each flexible node. The detected events are wirelessly transmitted to a base station, directly, as well as forwarded by other on-body nodes. For each node, a dual-polarized textile patch antenna serves as a platform for the flexible electronic circuitry. Therefore, the system is particularly suitable for comfortable and unobtrusive integration into garments. In the meantime, polarization diversity can be exploited to improve the reliability and energy-efficiency of the wireless transmission. Extensive experiments in realistic conditions have demonstrated that this new autonomous, body-centric, textile-antenna, wireless sensor network is able to correctly detect different operating conditions of a firefighter during an intervention. By relying on four network nodes integrated into the protective garment, this functionality is implemented locally, on the body, and in real time. In addition, the received sensor data are reliably transferred to a central access point at the command post, for more detailed and more comprehensive real-time visualization. This information provides coordinators and commanders with situational awareness of the entire rescue operation. A statistical analysis of measured on-body node-to-node, as well as off-body person-to-person channels is included, confirming the reliability of the communication system.

## 1. Introduction

Every year, more than 100 European firefighters lose their lives whilst saving others, as stated on the website of the Smart@Fire Seventh Framework Programme (FP7) project (2012–2015) [1]. Wireless sensor networks with on- and off-body wireless communication capabilities, detecting events by means of computationally efficient situational awareness algorithms, are very important to remotely monitor rescue workers and their environment. This functionality improves their safety and security, as well as the coordination of rescue operations, in general.

For critical applications, such as intervention by emergency services, novel fully-flexible and networked wearable systems must be developed, which can be unobtrusively and comfortably integrated into protective garments. A proof-of-concept of a smart textiles-based monitoring and coordinating system was developed earlier, with a special focus on firefighters, within the context of the ProeTEX FP7 project [2], whereas the ongoing Smart@Fire FP7 [1] project targets the further development of a realistic system. Electronic system integration on a flexible platform is essential, in order to design fully functional autonomous wearable sensing and communication networks, enabling automatic distributed event detection close to the sensors. Recent developments in single-chip multifunctional wireless transceivers enable the development of very compact, versatile, low-cost and low-power sensor network nodes. The integration of such a sensor system into professional clothing or patient garments, by means of textile antennas, maximizes user convenience, without the need for user interaction. However, until today, no garment-integrated fully-operational system is available.

In this paper, we present a novel, fully-autonomous, wearable, wireless sensor network. The network is composed of wirelessly interconnected flexible textile nodes that can quickly and unobtrusively be integrated inside the garments of a team of rescue workers. Each node autonomously performs cooperative synchronous acquisition and distributed event detection. Therefore, a low-power microcontroller on each flexible node implements computationally efficient situational-awareness algorithms that detect the events. In addition, sensor data and events are wirelessly transmitted to a base station and to the other on-body nodes. To set up reliable communication links, a dual-polarized textile patch antenna serves as a platform for the flexible electronic circuitry. A significant antenna gain [3,4] and large radiation efficiency, as well as effective shielding of the body from the radio-frequency energy is provided, thanks to the ground-plane antenna topology.

Wireless transmission of sensor data in indoor environments is often compromised by multi-path radio propagation, causing severe signal fading. It is well known that antenna diversity mitigates the effects of signal fading. Spatial and pattern antenna diversity techniques are exploited by distributing nodes over the body, separating them physically from each other. Additionally, the nodes presented allow polarization diversity, thanks to the dual-polarized patch antenna combined with the two-port transceiver chip.

The wearable network node is based on the physical-layer communication unit, of which the wireless channel behavior is described in [5]. In the further research presented here, this node is applied as a building block of a complete, highly power-efficient wearable wireless sensor network. Therefore, dedicated embedded software was developed, implementing a highly robust wireless on-body network protocol, performing synchronous data acquisition on different sensor nodes. As each node is equipped with an integrated three-axis accelerometer, the system is highly relevant for fall-detection applications [6]. Sensor fusion can be exploited by designing additional sensors into the node circuit in a straightforward manner.

Every network node includes a situational awareness algorithm, to detect and classify events autonomously or in cooperation with other nodes. Measurements are preprocessed on each node and wirelessly transmitted to a central access point. The situational awareness algorithms are computationally optimized to be executed on a low-cost and low-power microcontroller. More computationally expensive tasks can be performed at the base station, if necessary. Measurements confirm efficient synchronous operation for a four-node on-body system, as well as for a three-node person-to-person configuration. Synchronously acquired quadruple three-axis acceleration data are measured and transmitted, enabling detailed and real-time remote analysis of rescue-worker body orientation and movement.

In the literature, textile antenna research has mainly focused on dedicated antenna design and body-centric channel behavior, as well as on the performance of diversity textile antenna platforms [4,7,8]. In terms of complete on-body electronic systems, many rigid wearable nodes exist, often hindering the wearer's movements [9–12]. Many others also performed research on off-body antennas or body area radio propagation for sensor networks [13,14]. Ongoing integrated circuit design, intended for body area networks in the 2.45 GHz industrial, scientific and medical (ISM) radio band, is described in [15], indicating the relevance of this band for such applications. State-of-the-art, extremely low-power designs [16] leverage energy-harvesting sensor units for continuous long-term monitoring. A system relying on multiple wireless nodes, equipped with a number of sensors, fixed at several on-body locations, avoids false positive detections [17]. Network protocols and scheduling for body networks are discussed in [18–20]. Recent publications document wearable textile systems for biomedical monitoring [21]. However, in [21], the system does not provide network functionality, as no transmit function is available. On-body integration is impossible in that stage of development, as elaborate additional hardware is necessary for processing the received signal. Sampling of the unprocessed RF signal occurs via an external FPGA module, connected to a PC via USB. This system is suitable for lab experiments in a static setup only. Although the research presented in [22] is related, our system consists of fully wireless, flexible units, comfortably integrated into the rescue worker's garment. Signal-strength information is available for each data packet, providing valuable additional information for sensor localization [17] and body position recognition [23]. Processing of the measurements enables signal classification [24], with applications, such as gait analysis [25–27], kcal expenditure or physical rehab assessment [28]. The microcontroller in our proposed system executes a situational awareness algorithm on each on-body node, enabling automatic event detection and reporting by means of the wireless radio link.

We present a fully functional wearable sensor network employing a network protocol specifically developed for the difficult radio propagation environment encountered when performing indoor operations. To our knowledge, this is the first body-centric wireless wearable textile sensor network incorporating all of these features into a fully flexible garment-integrated system. Moreover, the system is fully tested in multiple measurement scenarios. The sensor data and their classification, as well as the radio propagation aspects are extensively documented in this paper. In the literature, no such system, documented and validated to a similar extent, was found.

Section 2 provides an overview of the system at the network level, outlining the proposed transmission protocol for on- and off-body communication. A description of the hardware composing each wireless node, being the dual-polarized antenna and key circuit components, as well as the proposed on-board computationally-efficient activity recognition algorithm, is given in Section 3. In Section 4, a measurement setup with a sensor-equipped test person is discussed, including a floor plan of the test environment and a graphical representation of the unprocessed measurement results. The relation between situational-awareness information, being the movements of the test person and his/her position in the floor plan and the sensor data, providing timestamps for the acceleration and received signal power data streams, is documented in Section 5. An evaluation of the on-body node-to-node link reliability is presented in Section 6, whereas an additional measurement assessing the performance of person-to-person communication in a three-person network is described in Section 7. A discussion follows in Section 8. The conclusions are summarized in Section 9.

## 2. System Overview

Patients, healthcare professionals and rescue workers should be monitored by a distributed sensor network, in order to assist caregivers and intervention coordinators. In such a network, data processed near the sensors are transmitted to a central access point. The proposed wearable wireless body sensor network was designed, constructed and validated in order to fulfill these requirements.

**Figure 1.**
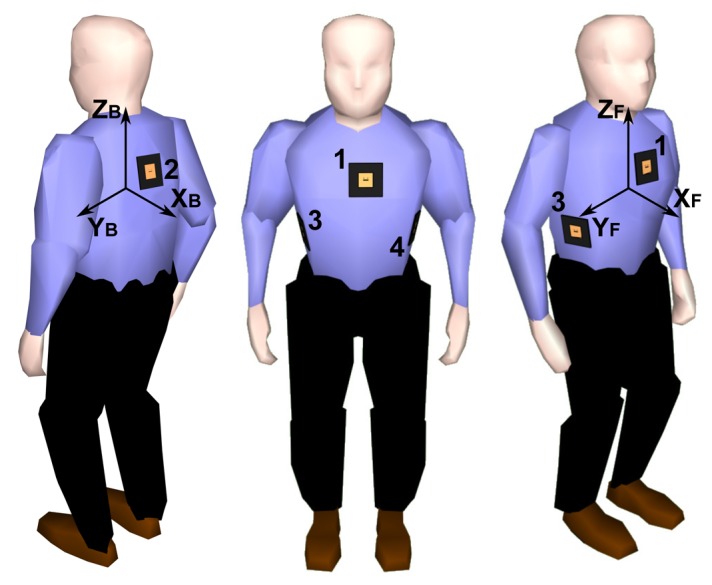
Wearable wireless body sensor network composed of four fully-autonomous and wireless textile nodes, synchronously capturing, preprocessing and relaying sensor data to a central access point.

The required functionality is provided by a dedicated network protocol embedded in each textile node's microprocessor, as well as in the central access point. The protocol is specifically designed for wireless communication between members and the coordinator of a small intervention team. In the typical scenario of a firefighting invention, for example, a group of two to three firefighters will penetrate a burning building, closely followed by a coordinator/commander outside the building. The envisaged network protocol shall ensure reliable communication and detect events within the team. Moreover, the protocol must be able to deliver this information to the command post, which may issue alerts, all without significant delay. We first outline this protocol, designed for an N-node body-worn network, involving multiple team members and the coordinator. In the next section, we proceed to a detailed description of the hardware components that compose each textile node of the body sensor network shown in Figure 1.

### 2.1. Network Protocol for Synchronous Measurements

**Figure 2.**
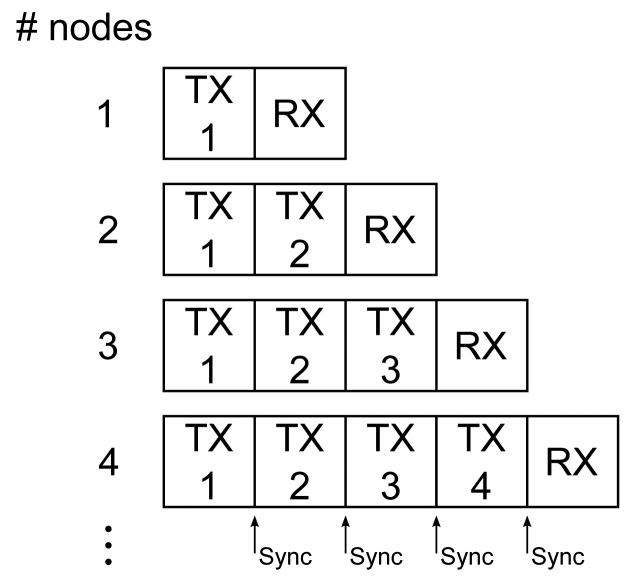
Power-on cycle and time-slot structure of the autonomous body-centric network.

**Figure 3.**
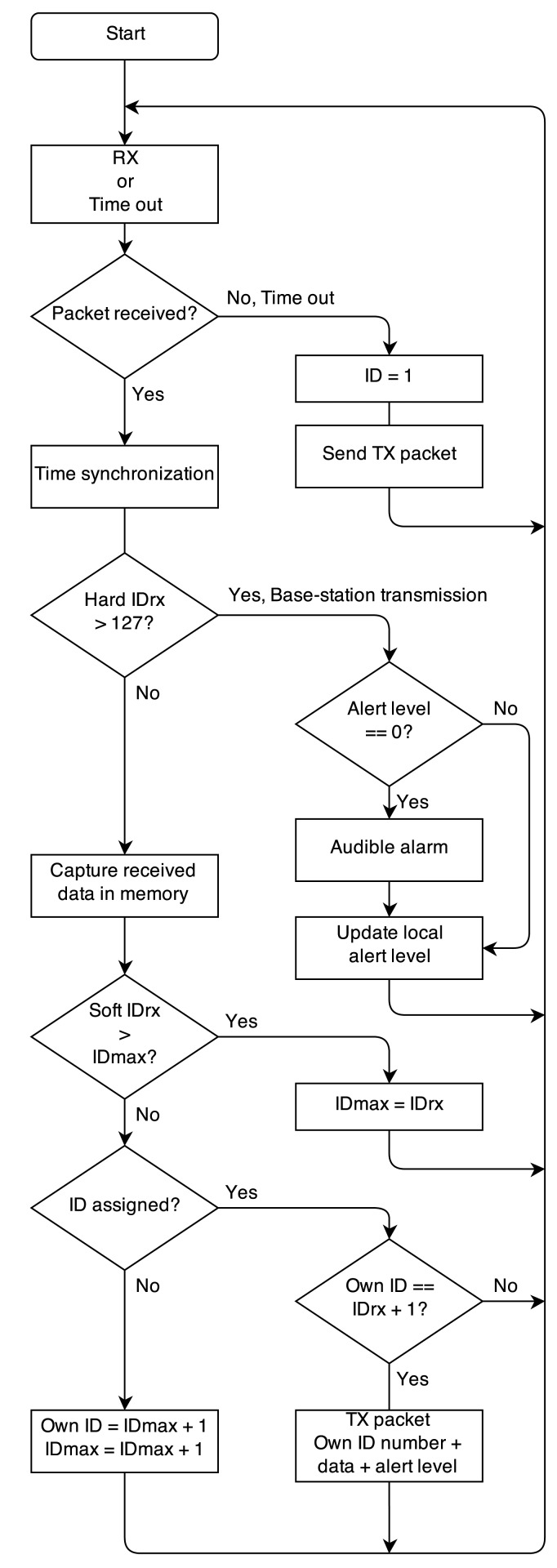
Simplified flowchart of the network protocol.

**Figure 4.**
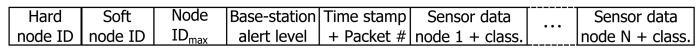
Data packet structure.

Synchronous sensor data acquisition, including a wireless communication link to a central access point, is implemented for an N-node body-worn network by means of the following protocol:

- Power-on cycle and node enumeration: During subsequent manual power-on of the nodes, the nodes detect each other's presence in the network and are automatically enumerated, acquiring their unique soft-ID's, which determine their time slots in the transmission cycle. Nodes have to be switched on sequentially, respecting at least one second in between the manual switch actions. Please note the proposed system is composed of battery-operated truly wireless sensor units, leading to a sequential power-on cycle due to the absence of a common power supply or any other wired connection between the nodes.
  – When powered on, each node listens for packets from other nodes. If no packets are received within a one-second time-out period, this node is assigned soft-ID = 1 and autonomously starts transmitting sensor data, including this soft-ID number.
  – After the last transmitted packet of the transmission cycle, an extra receive time slot is preserved to listen for other packets from new nodes that can join the network, as displayed in Figure 2.
  – When a next node is powered-on, the new node first listens for data packets transmitted by other nodes, receiving data packets from all active nodes. The new node responds with its own sensor data and dynamically determined soft-ID number (being the highest soft-ID number in the network +1). This step is repeated for all N nodes in the body-centric network.
  – Given the successful reception rate of more than 95% observed in our measurements (as further documented in Section 6), the probability of missing all packets in the time-out period is extremely low, successfully avoiding an erroneous multiple assignment of the same soft-ID number. In practice, the network activity and, hence, the enumeration process are also monitored and verified by the base station, confirming a correct initialization. The power-on cycle and enumeration process should be performed by all firefighters and the coordinator operating the base station well within each other's range, which is a realistic scenario, as firefighters always enter a building as a small team.
  – Each node also transmits a unique and fixed hard-ID, allowing unambiguous identification by the base station, as well as avoiding re-enumeration of already active nodes in case of communication errors.
  – When all nodes in the body-centric network are active, a time-slot is kept available to receive base station transmissions, as shown in Figure 2 for a four-node network. Base-station transmissions are recognized by a hard-ID >127 and do not lead to further network enumeration. In case of an emergency, the base station transmits a data packet containing an alert level. The alert level initiates different alarm conditions at the rescue worker's side. When an alert level is received by one or more of the wireless nodes, the alert level is forwarded by the wireless nodes themselves in their own transmit time slot together with the sensor data, to ensure all rescue workers receive the alert message. The alert level is stored on each wearable node. The initial alert level is zero; higher alert levels lead to audible alarm signals on the wearable nodes via a beeper, which has to be connected to a microcontroller output pin reserved for this purpose.
- Data forwarding and measurement synchronization are possible thanks to the network nodes continuously monitoring each other's transmissions. At each time instant, the sensor data from the currently transmitting node are stored in the memory of each receiving node, which will forward the collected data in its own transmit time slot. Thanks to the data forwarding, a redundancy leading to a performance increase comparable to N-th order transmit diversity gain is obtained, providing a highly reliable cooperative data link towards the base station. The nodes synchronize their clocks using the time stamps of the received data packets from the other nodes. This enables fully-synchronous sensor data capturing on all nodes. The flowchart of the network protocol is shown in Figure 3.
- Data packet structure: The sensor data captured by the nodes are organized into a packet, as shown in Figure 4, transmitted by employing the IEEE 802.15.4 mode supported by the transceiver chip, automatically adding an error-detecting code, ensuring the correctness of the received data. The node's hard-ID corresponds to a unique fixed serial number for each physical node, whereas the soft-ID number was assigned automatically during the network enumeration process.
- Scalability: The platform is highly versatile and can also operate in larger networks.
  – Up to 12 nodes can actively forward packets from the base station or other users in the network. These nodes can be distributed over the members of an intervention team; three firefighters wearing four nodes or six firefighters wearing two nodes are efficient configurations. Two nodes per body provide enough information, but four nodes provide more accuracy and reliability thanks to the redundancy. Authorities in the firefighting world [1] state that teams of three firefighters are the maximum to be monitored by one commander.
  – Extra time slots can be provided in the transmission protocol to enable dual-polarized diversity reception, by sending all transmitted data packets twice and receiving odd and even packets alternatingly on different polarizations.
  – Parallel networks can be deployed at different frequencies; their data is then combined at the base station, using multiple low-cost receivers. In IEEE 802.15.4 mode, the 2.45 GHz I.S.M.-band provides 16 channels.

## 3. Wireless Sensor Node Implementation

### 3.1. Hardware Description

**Figure 5.**
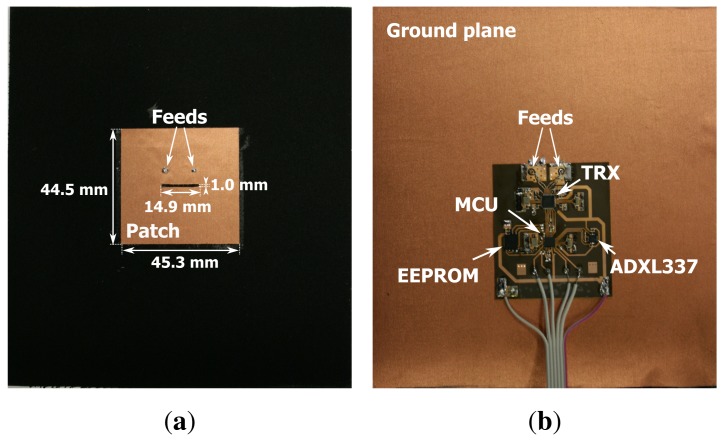
(**a**) front side of the wireless sensor node; (**b**) back side of the wireless sensor node with the electronic components mounted on the flexible substrate.

**Figure 6.**
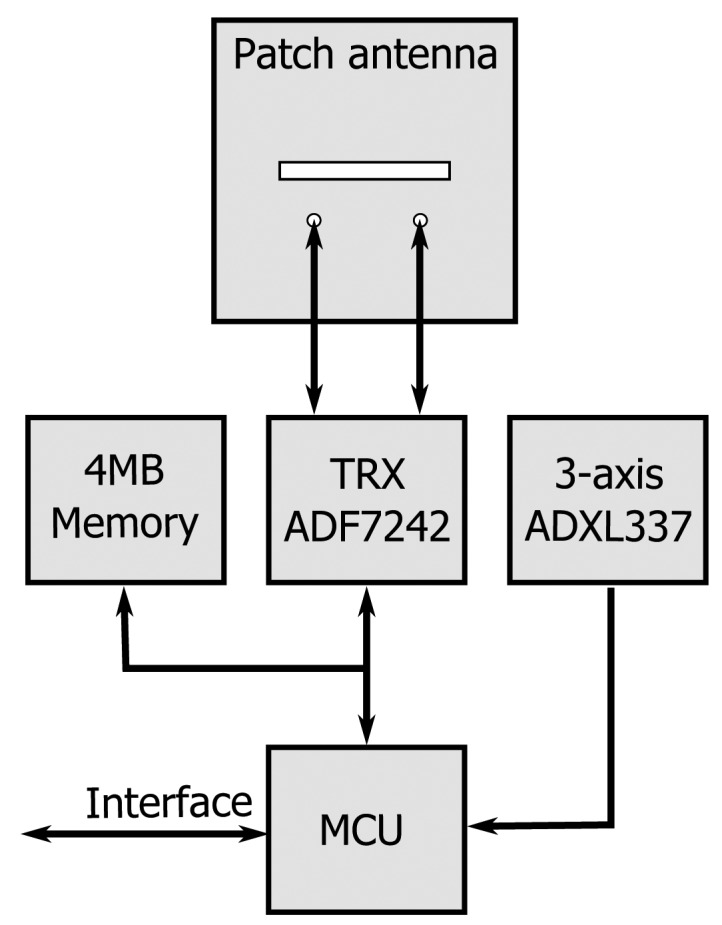
Block diagram of the wearable node, showing the MCU, flash memory, accelerometer and transceiver chip connected to the textile patch antenna.

Figure 5a,b shows a prototype of an autonomous wireless sensor node completely integrated into a flexible garment-integrated patch and powered by a single battery. In Figure 5a, the front side of the wireless sensor node is shown, where the patch antenna is visible with its dimensions. Figure 5b gives an overview of the backside of the wireless sensor node; this side includes all of the electronic components mounted on the flexible substrate. We now discuss the various components composing the block diagram of the node in Figure 6.

**Figure 7.**
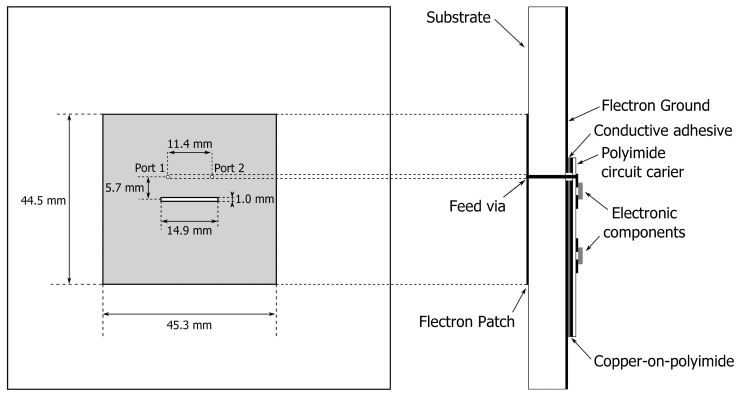
Cross-section of the dual-polarized textile antenna with electronic circuitry mounted onto the feed plane.

The basic platform for the wireless node is a dual-polarized textile patch antenna, as shown in Figure 7. Details about this antenna and its performance can be found in [4]. This compact wearable antenna is fully breathable, flexible and includes two feeds, enabling the excitation of two orthogonal linearly-polarized waves, with an antenna gain of 6 dBi along the boresight and a better than 15 dB isolation between the feed ports. The textile material makes the unit flexible and lightweight, without losing antenna performance, in comparison to rigid antennas. The antenna ground plane is constructed using FlecTron, a low-cost, conductive, electro-textile material with a thickness less than 0.25 mm and a surface resistivity less 0.1Ω/sq., minimizing the influence of the body in close proximity to the antenna. The substrate material is closed-cell, flexible, expanded-rubber protective foam, commonly used in protective garments for rescue workers (density = 187.3 kg/m^3^, permittivity *ϵ_r_* = 1.53 and tan *δ* = 0.0012) with a thickness of 5 mm. The flexible foam will help to protect the electronic circuitry from external factors, such as humidity. As the networking-enabled wearable node designed here presents a further development of the wireless textile transceiver documented in [5], we refer the reader to this text for details about its fabrication, together with a validation of its physical-layer wireless communication performance, employing polarization diversity.

On the wireless node, a variety of analog and digital sensors can be incorporated. In this paper, we discuss the integration of an Analog Devices ADXL 337 three-axis accelerometer into our prototype. This sensor is very compact and provides accurate acceleration measurement data, which will be preprocessed, locally interpreted and transmitted to a central access point. The wireless sensor node measures acceleration with a specified full-scale range of ±3 g. Acceleration caused by gravity allows sensing of body orientation, whereas accelerations resulting from motion, shock or vibration produce information about the firefighter's actions. The accelerometer sensor is very robust as, according to the data sheet, 10,000 *g* shock survival is guaranteed [29].

The microcontroller forms the heart of the system, providing the distributed network functionality. A highly compact and low-power advanced single-cycle microcontroller, the Silicon Laboratories C8051F921, is used. This processor collects sensor data and organizes it into packets for wireless transmission or storage into memory. Embedded software for this controller is developed in the C programming language and uploaded into the controller's nonvolatile code memory by the In-System Programming interface via a USB cable [30].

On-board flash memory is available as nonvolatile storage space for measurement data. The memory unit is used as a buffer or as a data storage for processing and analysis. At a rate of 25 measurements per second, at least 11 h of continuous three-axis accelerometer data can be stored in the 4 MB flash installed on the prototype.

The state-of-the-art Analog Devices ADF7242 wireless 2.45 GHz ISM-band transceiver is used to set up the wireless data link. This is the first single-chip 2.45 GHz transceiver incorporating diversity, as well as IEEE 802.15.4 and GFSK modulation. The maximum output power of the ADF 7242 is limited to +4.8 dBm, which is well within the limits imposed by regulations (20 dBm, ETSI standard EN 300 328 [31] for wide band transmissions, such as in the IEEE 802.15.4-2006 mode). If desired, the range of the nodes can be significantly extended by increasing the transmit power up to the legal limit of +20 dBm. This option is discussed in the datasheet [32] of the ADF7242 transceiver chip and involves designing an integrated RF amplifier into the circuit.

### 3.2. Computationally Simple Classification

**Figure 8.**
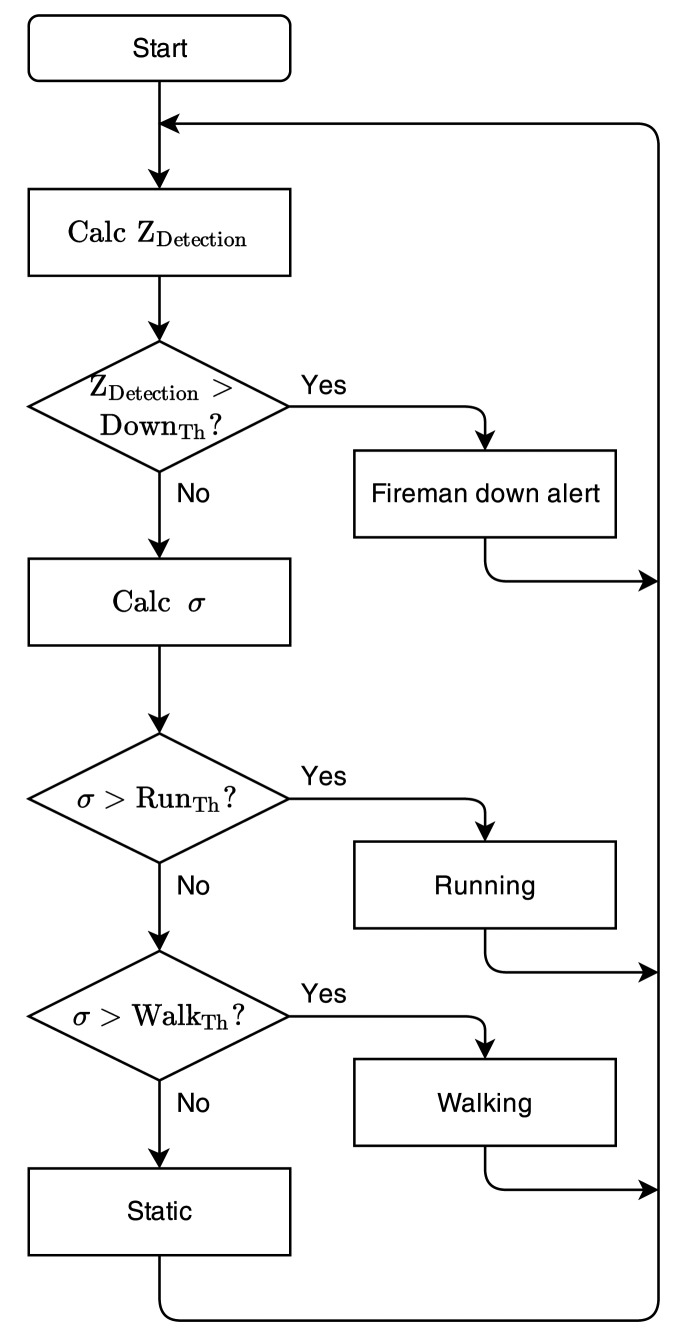
Flowchart of the computationally simple classification.

Each node includes a three-axis accelerometer, providing three independent sensor data streams. The sensor data can be used, independently or in cooperation with sensor data of the other nodes in the on-body network, to implement real-time activity recognition. An algorithm is proposed to detect and classify activities performed by firefighters during rescue operations, optimized for implementation on the microcontroller of each sensor node, with minimal processing power. Existing systems, as documented in [10], do not implement signal classification and event detection on the node itself. A flowchart of the computationally simple classification is given in Figure 8.

The most important situation that needs to be monitored in the rescue-worker application is lying down [2], as this potentially corresponds to an emergency situation where the rescue worker's life is at risk. When the person is walking or standing, the measured gravity vector value will always be approximately −1 *g* along the *z*-axis. When lying down, the measured gravity vectors for all axes reorient, and no repetitive accelerations are detected. Lying flat on the ground, the gravity vector for the *z*-axis will be approximately 0 *g*. By combining the measurements along the *z*-axis from all nodes, averaged over one second of sensor acquisition, lying down is readily detected. The decision rule for lying down is given by:

(1) $$\langle Z_{\text{detection}}\rangle =\frac{\overset{N}{\underset{n=1}{\text{∑}}}\overset{M}{\underset{m=1}{\text{∑}}}Z_{n,m}}{N\cdot M}>{\text{Down}}_{\text{Th}}\to \text{Lying down}$$

with *n* the node number, *N* the total number of nodes, *m* the measurement sample number and *M* = *f_s_*.Δ*t* = Sample rate. 1*s* the number of measurements per node during a one-second time window. The threshold is set to Down_Th_ = −0.5 *g*, which corresponds to the firefighter lying down, with an angle of 30° or less (straight up = 90°) with respect to the ground.

A detection algorithm is proposed for walking or running, based on the repetitive accelerations observed in [27,33]. Running generates larger and higher-frequency accelerations, resulting in a larger standard deviation of the accelerometer data, compared to walking [25-27]. Therefore, the standard deviation *σ* of the accelerometer sensor data is observed over a one-second time frame, given by:

(2) $$\sigma =\sqrt{\frac{\overset{M}{\underset{m=1}{\text{∑}}}{\left(\bar{Z}-z_{m}\right)}^{2}}{M}}$$

with *M* = *f_s_*, being the sample rate, and *Z̅* the average over a one-second time window. The decision rules for walking and running are given by the following thresholds:

(3) $${\text{Run}}_{\text{Th}}\geq \sigma >{\text{Walk}}_{\text{Th}}\to \text{Walking}$$

(4) $$\sigma >{\text{Run}}_{\text{Th}}\to \text{Walking}$$

When *σ* exceeds a preselected threshold level, the corresponding activity is in progress. The threshold values for the activity algorithm are empirically selected:

(5) 0.4g≥σ>0.08g→Walking

(6) σ>0.4g→Running

When an alarm situation occurs, the status of the activity recognition algorithm is transported in the wireless data packet to the base station, directly or with priority, and forwarded by other nodes in the network, providing a fast and very reliable information link from the rescue worker to the base station.

## 4. Measurements for Four On-Body Nodes

The performance of the wireless sensor network is assessed for a realistic application, where the wearable system is deployed in a protective garment, worn by a rescue worker performing a number of movements and postures at different locations. A number of real-world situations are studied in an indoor office environment, as well as in an outdoor scenario.

### 4.1. Measurement Setup

Four wireless nodes are integrated into a firefighter garment, deployed on the front, back, left and right sides of the body. The antennas radiate away from the body in different directions, providing spatial, as well as pattern diversity. The setup is illustrated in Figure 1. For clarity, only the front (1) and rear (2) nodes are initially taken into consideration. Given the relative orientation of the two nodes, the vectors of the *x*- and *y*-axes of both nodes will be opposite. The gravity vectors provide information about the orientation of the body of the rescue worker. The central access point is represented by a receiving node that relies on similar hardware as the mobile nodes, but now with two omni-directional monopole antennas connected to the inputs.

**Figure 9.**
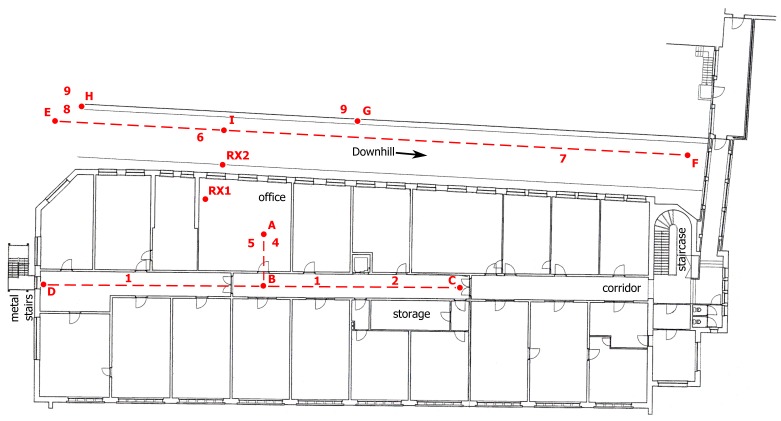
Floor plan of the office environment at Ghent University.

In the indoor office environment, the base station node is located at a height of 2 m above the office floor (RX1) and connected to a computer performing real-time data processing. The office at Ghent University is located on the first floor, consisting of solid brick floors and reinforced concrete walls. The floor plan of the office and its surroundings is given in Figure 9. Next to the building, there is a inclined street with its down-hill direction to the left, reaching the ground floor level at the right, surrounded by other buildings. In the outdoor environment, the receive node is placed outside the office building (RX2), along the inclined street, as illustrated on the floor plan in Figure 9.

### 4.2. Data Reliability

In the course of an indoor measurement, where the firefighter walks in the office floor along the path A–B–D–B–C–B–A in the floor plan, as shown in Figure 9, a total of 16 packets out of the full set of 1900 packets transmitted to RX1 was lost for four nodes. However, as the sensor data pertaining to the nodes from which packets were missing at the base station were repeated by the other three on-body nodes, all sensor data were recovered. In an indoor environment, the signals from *N* different nodes are influenced by decorrelated fading, leading to an *N*-th order diversity gain. This clearly demonstrates the vast improvement in transmit reliability of the body-centric network. An overview of the packet loss and recovery is shown in Table 1.

### 4.3. Synchronization of Four Nodes

**Figure 10.**
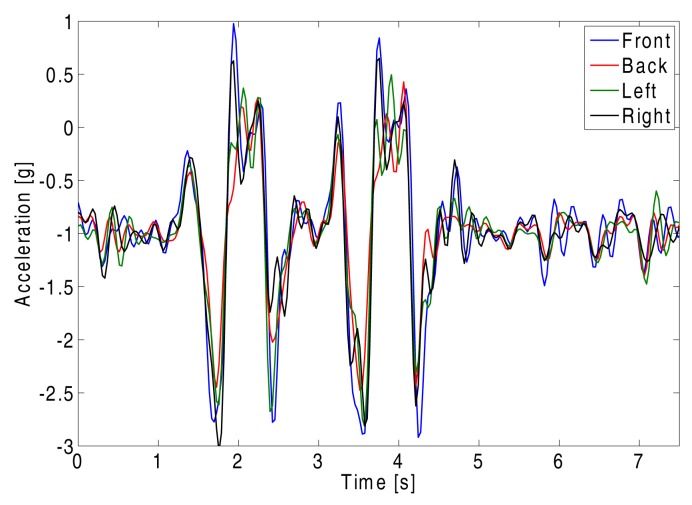
Synchronization of the sensor data related to the vertical z-axis of the wireless nodes, when the test-person is jumping.

In Figure 10, the sensor data, acquired along the vertical accelerometer axes during a jump, are illustrated, in order to demonstrate the synchronicity of the measurements on all four nodes. Traces for the other axes are also synchronized, but have been omitted for the sake of graph clarity.

### 4.4. Accelerometer Measurement

**Figure 11.**
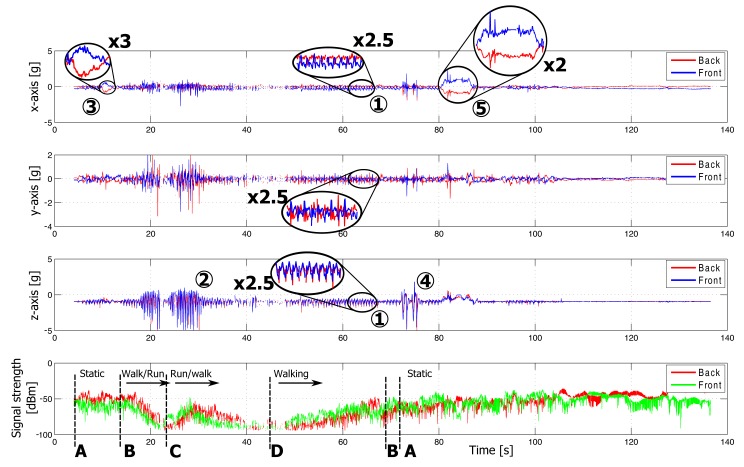
Measurement results along the indoor path A–B–C–D–B–A, followed by some activities performed in the neighborhood of A (*x*, *y*, *z*-axes + signal strength).

**Figure 12.**
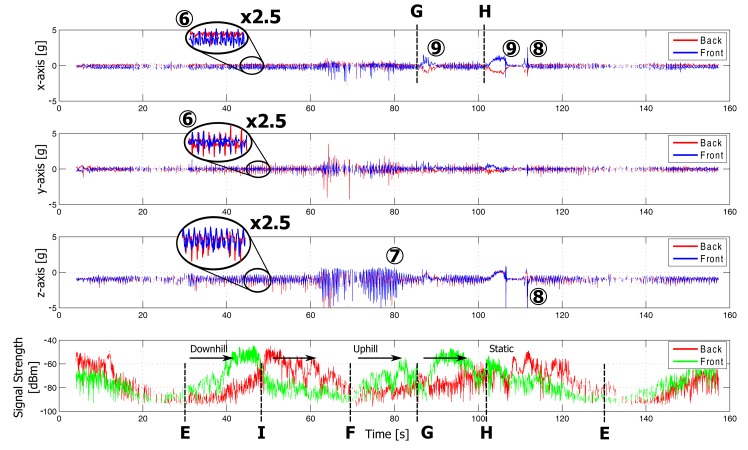
Measurement results along the outdoor path, starting with random walk moving to location E, followed by the path E–I–F–G–H, ending with some activities performed in the neighborhood of H and random walk around location E (*x*, *y*, *z*-axes + signal strength).

Plots of the measurements in both indoor and outdoor environments are displayed in Figures 11 and 12, respectively. Features visible in the graph are caused by specific movements and postures of the rescue worker. They are linked to his position in the floor plan, as shown in Figure 9. For clarity of the graphs, the sensor data for only two nodes are shown. The on-body network provides reliable connectivity over the full trajectory covered by the test person. Further analysis of the sensor data follows in Section 5.

### 4.5. Signal Strength Measurements

While the test person walks along the outdoor path E–F, the receive node RX2 at the base station observes the signal power of the received packets from the front and back node. At the beginning of the path (E, marked on Figure 12), the front node is in Line of Sight (LoS) with the receiver node, providing a large signal strength on the receiving node. When the test person passes by the fixed node at Position I, the signal strength of both nodes are equal. After this point, the back node is in LoS with the fixed node, providing a large signal strength in comparison with the front node. Differences in signal strength up to 35 dB occur between the two nodes. The configuration of a front/back sensor node system has clear advantages compared to a single sensor system. The total coverage area of the sensor system increases thanks to the transmit diversity gain and the multi-hop wireless network topology.

### 4.6. Power Consumption

The wireless node is powered by a small (5 mm × 25 mm × 35 mm) one-cell Lithium polymer (Li-po) battery of 400 mAh and a low-drop linear voltage regulator. From the technical data sheet of the various integrated components of the wireless sensor, an estimation of the power consumption can be made. The microcontroller will consume an average current of 4 mA at 3.3 V and at a clock frequency 24.576 MHz. In sleep mode, the current consumption can be lowered to 600 nA. The accelerometer used on the wireless sensor only consumes 0.3 mA during operation at 3.3 V. The flash memory consumes on average 12 mA at 3.3 V while operating (reading or writing); in standby mode, the current consumption is lowered to 25 μA or to 5 μA in deep power-down mode. The most power consuming device on the wireless sensor is the transceiver chip. At the the highest output power, a maximum current of 25 mA is used at 3.3 V. In receiving mode, the maximum current consumption is 19 mA. While the transceiver is not transmitting data packets, it will be configured in the receiving mode. In idle mode, the power consumption is lowered to 300 nA. An overview of the current consumption is summarized in Table 2.

The maximum current consumption is estimated at 29.4 mA at 3.3 V, taking into account the maximum power consumption while transmitting and no operation of the flash memory is performed. Due to the circuit topology, it is not possible to read or write data into the flash memory while transmitting a data packet.

In full operation, the measured average power consumption of one node of the wearable sensor network equals 90 mW (27 mA current consumption, at 3.3 V supply voltage) with negligible variation when operating in transmit or receive mode. This enables the sensor network to operate for many hours, without the need for charging the battery. Furthermore, the power consumption can further be minimized by employing the sleep mode of the system when there is no need to operate continuously at high speed. In this mode, the total power consumption is less than 10 mW. The system can be activated at regular intervals based on the hardware wake-up timer integrated in the microcontroller, to check the activity by the base station or other on-body nodes.

## 5. Spectrogram and Classification of Accelerometer Measurements

The sensor data from the four-node on-body experiment is now analyzed employing classification algorithms. Activity recognition is illustrated at the base station, as well as locally at each wearable node.

### 5.1. Classification of the Accelerometer Data

The following actions are clearly detectable based on the accelerometer measurements. The markers refer to floorplan in Figure 9, for the location of the firefighter and Figure 11 or 12 for the corresponding sensor data.

- Walking (1, 6): A firefighter walks at moderate speed. This movement introduces significant repetitive accelerations measured by both wireless nodes. The accelerations are clearly visible along all of the axes of the sensors, as shown in Figures 11 and 12. The walking speed is determined based on the repetition frequency of the accelerations, allowing step counting.
- Running (2, 7) introduces stronger accelerations on both wireless nodes. These accelerations are clearly visible in Figures 11 and 12 and are easily detected based on the acceleration values and main frequency component.
- Bending (3) of the rescue worker introduces reorientation of the measured gravity vector caused by the movement of the upper body. Thanks to the opposite direction of the nodes on the body, the signs of the accelerations measured on the front and back are opposite, allowing easy detection of bending. This movement is performed at Point A in the office and is clearly detectable in the measurement data in Figure 11. A difference of the opposite gravity vectors (larger than 0.5 *g*) indicates bending.
- Jumping (4, 8) causes large accelerations along all axes of the accelerometers, especially along the z-axis of the test person, as seen in Figures 11 and 12. The test person climbs onto a wall and jumps down at Point H. In the indoor environment, the test person jumps up and down at Point B.
- Lying down (5): When a person is lying on the ground, the accelerometer will determine the direction of the gravity vector, providing body orientation data. In the measurement, the test person lies face-down at Point A in Figure 11, causing both sensor nodes to provide oppositely orientedgravity vectors along the x-axis, with a maximum difference of the opposite gravity vectors of 2 *g*. The gravity vectors for both nodes along the z-axis will approximate 0 *g*. When a position occurs in between frontal and sideways lying, the difference of the opposite gravity vectors is still detectable along both the *x*- or *y*-axes. A continuous difference of at least 1.4 *g* along the *x*- or *y*-axes is observed, clearly indicating this lying position.
- Climbing (9) on top of objects also introduces a reorientation of the measured gravity vector. This movement consists of lateral (along the *y*-axis) and forward (along the *x*-axis) bending of the body, together with a reorientation of the gravity vector along the *z*-axis. The test person climbs onto a wall of approximately 1.5 m and 1 m height at Point G and Point H, respectively.

### 5.2. Spectrogram

**Figure 13.**
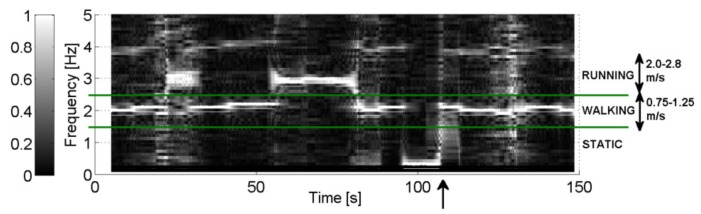
Spectrogram for the frequency components of the accelerometers in the outdoor measurement (normalized amplitude).

In Figure 13, the rectangular windowed fast Fourier transform (FFT) of the accelerometer data is plotted for Node 1, as a spectrogram with a time window of 256 samples, clearly indicating different movements of the test person. This spectrogram is normalized to the amplitude of the largest frequency component.

At a normal walking speed, a main frequency component of 2 Hz is observed, whereas 3 Hz is obtained when running. A person who is running takes larger steps, which explains why an approximately double speed results in an increase of the main frequency component by only a factor of 1.5. However, running is easily detected, because both the frequency and amplitude of the main spectral component significantly increase compared to walking. In a stationary position, a frequency component of 0 Hz (DC), is observed. This is clearly shown when the test person has climbed on the wall in the outdoor measurement. Accelerations for step counting are easily detected in the spectrogram. After the test person climbs the wall, the increasing main frequency component in the time needed to achieve normal walking speed is indicated by an arrow in Figure 13, illustrating the accuracy obtained by the sensor network.

### 5.3. Activity Recognition Results

**Figure 14.**
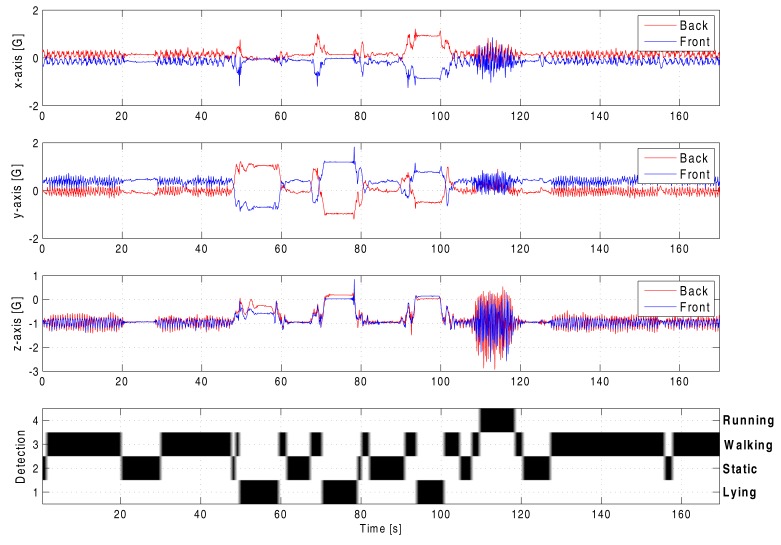
Activity recognition results for walking, running and lying down in different positions.

In Figure 14, the measurement data from two nodes are displayed (for clarity reasons), while several activities are performed during a time frame of approximately 3 min. Below the acceleration data from the two sensor nodes, the status of the activity recognition algorithm is shown. The implementation of the algorithm allows easy recognition of the current activity of the rescue worker. Four levels are used in the graph to indicate the status of the activity recognition algorithm.

As can easily be verified in the unprocessed measurement in Figure 14, the classification algorithm successfully determines the user state in a computationally efficient way. Although detection on one on-body node is already remarkably accurate, the combination of data from all sensors in the on-body network further increases the reliability as a classification system for user actions.

## 6. Evaluation of On-Body Node-to-Node Link Reliability

An important aspect in the functionality of the body-worn system is that off-body transmissions are employed for the node-to-node communication on the same body, relying on reflections in the environment. As this issue raises questions about link reliability, especially between front and back nodes, an experiment is performed illustrating the received signal levels, as well as the packet loss for the node-to-node links.

A person equipped with four nodes is performing a random walking behavior in an indoor environment, including maneuvers, such as jumping and lying down. The nodes numbered 1 to 4 are located in the front, back, left and right of the body, in that order. The base station is placed within line of sight of the walking person. The total number of 5940 received packets corresponds to 4 min of walking and allows accurate statistics of the link behavior.

**Figure 15.**
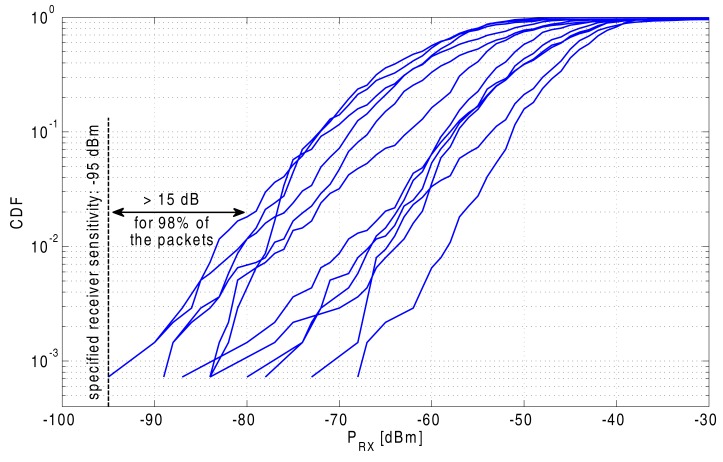
Cumulative distribution function of the received signal levels for the 12 on-body node-to-node links in a front, back, left and right configuration.

The cumulative distribution function of the received signal powers for the 12 node-to-node links is displayed in Figure 15. These curves illustrate that the signal level is at least 15 dB above the ADF7242 transceiver's specified receiving threshold of −95 dBm for 98% of the received packets.

In total, 12 node-to-node links exist in a four-node network. The nodes corresponding to each link are listed in Table 3, including the median received power and packet loss for each link. The maximum number of subsequently lost packets on each link is included, as this is valuable information to acknowledge the reliability of the network enumeration protocol explained in Section 2.1.

The maximum number of subsequently lost packets on any node-to-node link is three, corresponding to a maximum link interruption of 120 ms, whereas the timeout for the enumeration process is 1 s. Note that packets are always received at a 25 Hz rate, independent of the number of active nodes, as transmissions by all active nodes are received, making the probability of an enumeration fault extremely low. In the unlikely case that such an error does occur, this will be immediately detected by the base station.

## 7. Performance Analysis for a Three-Person Network

**Figure 16.**
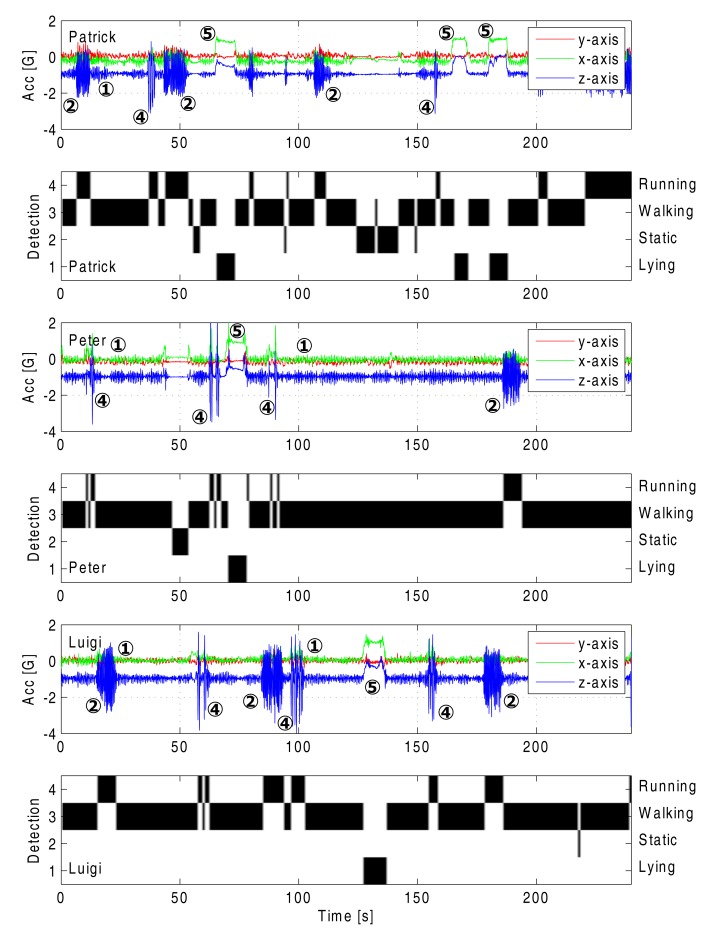
Accelerometer data and classification results for three persons in the wireless sensor network.

As a further assessment of the reliability of the wireless network and classification in the case of multiple persons, a measurement is performed employing three persons, each wearing one network node on the chest. The three persons are performing independent random walks within the same area, separated by varying distances of up to 20 m. The persons are all randomly switching between actions, such as walking, running, standing still, laying down and jumping, clearly illustrating the presence of separate persons. The computationally simple classification method is performed on the sensor data of the three persons, confirming its reliability for different test persons. Both the sensor data and the classification results are shown in Figure 16. The markers on the graphs refer to the actions, as described in Section 5. Although only one node is used for each person to measure accelerations and perform automatic classification, the computationally simple algorithm still performs very well.

Additionally, the experiment proves the following important communication properties of the system:

- Node-to-node communication is also very reliable between nodes mounted on different persons operating in a team.
- Thanks to the forwarding of packets, not a single measurement is lost, even when only one unit installed on each body.

**Figure 17.**
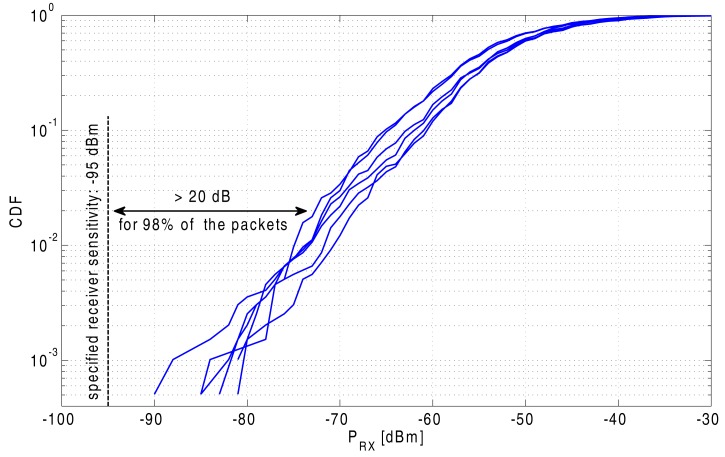
Cumulative distribution function of the received signal levels for the six off-body person-to-person links.

The Cumulative Distribution Function (CDF) of the received signal powers for the six node-to-node links is displayed in Figure 17. These curves illustrate that the signal level is at least 20 dB above the ADF7242 transceiver's specified receiving threshold of −95 dBm for 98% of the received packets. In comparison, the node-to-node communication is even more reliable between nodes on different bodies, compared to the link between nodes on the same body. This behavior is as expected, as when the nodes are mounted on different persons of a team a line-of-sight link often exists between these nodes.

Interestingly, the median received power is in the same range for the person-to-person links compared to on-body node-to-node links. The persons are constantly reorienting during the random walk and located at varying distances from each other, resulting in a constantly changing path loss. However, the spread between different CDF curves is much more limited for the person-to-person links. The reason for this behavior is that for the full measurement, the propagation conditions are similar for each pair of nodes, with each node worn on the front of a person performing a random walk over a 4-min time span. In comparison, for the on-body node-to-node communication, the nodes are in fixed locations on the same body, causing systematic differences in the CDF curves due to the specific set of fixed body locations corresponding to each pair of communicating nodes. The packet loss values, as shown in Table 4, are also significantly lower for the person-to-person links, confirming the results observed in the CDF characteristics.

We conclude that distributing the nodes over several persons does not compromise the wireless communication between nodes or the network functionality in any way. Node-to-node links on the same body, as well as person-to-person links are always very reliable, thanks to the low packet loss for each link, combined with the redundancy introduced by forwarding packets, leading to a reliability increase of the communication system comparable to *N*-th order transmit diversity for an *N*-node network [34].

## 8. Discussion

The flexible wearable wireless network system successfully measured three-axis accelerations and transmitted these measurements and a classification thereof to the base station without a single missing sensor measurement in all experiments performed. Additionally, this performance was achieved with very low delays, even when data was received via forwarding.

An important advantage of the node is surely the textile patch antenna, which offers more antenna gain than the popular inverted-F antenna seen in many other publications. Both the circuit and the antenna are very flexible, providing a great advantage for garment integration of the system. The dedicated network protocol and embedded classification algorithm are important extra features increasing the reliability.

Although a minor degree of packet loss, varying between 0.2% and 4.5%, does occur on the individual links between nodes, or between a node and the base station, a dedicated network protocol improves connectivity by means of data forwarding. In this network protocol, specifically designed for the difficult radio propagation conditions experienced by moving persons in an indoor environment, all data transmitted by each network node are forwarded by all of the other nodes. Data forwarding creates redundancy, resulting in a very reliable data transmission towards the base station, which results in a performance increase comparable to N-th order selection combining diversity for an N-node network, as shown earlier in [34]. This approach leads to a very significant increase in data reliability and/or communication range.

The proposed protocol is an important feature of the system. Thanks to its simplicity and the data redundancy introduced, it is very suitable for the typical scenario of moving persons in an indoor environment, experiencing dramatically varying radio propagation between each set of nodes or between each node and the base station. Established network protocols, such as ZigBee, used in many publications, are more suitable for networks with nodes in static positions. Problems arise when channels are rapidly changing, for each transmitted packet, due to the higher complexity of the protocol. Additionally, the proposed protocol allows quasi delay-free operation, thanks to the immediate forwarding of data. For a 12-node network, the delay would maximally augment to 0.5 s in the worst-case propagation conditions.

The network does require a power-on sequence to initialize. Nodes should be switched on subsequently with all persons wearing nodes well within each other's range and respecting a one-second interval in between switch actions. This disadvantage does not cause a problem in realistic interventions where the members of a small team start their actions together. Note that the protocol offers extreme reliability in return, after correct initialization. Many problems occurring with more complex protocols are avoided. Suddenly missing nodes, appearing again later, rejoin the network immediately, as if nothing happened. Of course, the proposed protocol is only suitable for low data-rate communication and is focusing on data integrity in exchange for data throughput. However, in realistic firefighting conditions, only low date-rate information is available, as high data-rate sources, such as video cameras, are unusable, due to smoke causing zero visibility.

Specific validation of on-body node-to-node links, as well as off-body person-to-person links was performed, resulting in the very important conclusion that either form of communication is very reliable. More than 98% of the transmitted packets were received at a power level that was at least 15 dB and 20 dB higher than the specified receiving threshold for the transceiver chip, for node-to-node and person-to-person links, respectively.

A classification algorithm was also programmed on the microcontroller on each node and validated in realistic conditions. The three-person measurement demonstrated the reliable operation of this computationally simple algorithm for persons of different body sizes. Despite the simplicity of the embedded classification, the algorithm combined with the forwarding of the classification results has the potential to provide information about the user state to the base station in extremely bad radio propagation conditions, where the base station is not receiving all of the raw measurement data anymore.

## 9. Conclusions

A novel autonomous wearable cooperative wireless sensor node network was developed, synchronously measuring and interpreting accelerometer data on multiple nodes and transmitting the data to a base station. Event detection is performed close to the sensors, by means of the on-node low-energy microcontroller, running a computationally-efficient algorithm. The system operates very reliably in various radio-propagation environments. The received signal strength can be used as a valuable additional parameter, providing ranging and orientation information. On-body node-to-node communication is exploited to synchronize measurements on multiple autonomous nodes, at different body locations, and to share sensor data between these nodes. N-th order transmit diversity performance is approached, by repeating the sensor data from the other on-body nodes, drastically enhancing communication reliability by eliminating packet loss. The system is highly valuable for rescue workers and law-enforcement officers, as well as for a wide range of military and civilian applications. Network functionality can readily be enhanced, by extending the software on the embedded microcontroller. Besides increasing the number of nodes on the body of one person, several persons can join the sensor network, share each other's sensor data and forward each other's data packets. Sensor fusion can be enabled by integrating multiple sensors, such as temperature, moisture and gas sensors, on a single wireless node. The small-size and low-cost sensor nodes, easily and comfortably integrated into clothing, also implement a real-time situational awareness system.

## Figures and Tables

**Table 1.** Overview of the packet loss and recovery during the measurement campaign.

**Parameter or Variable**	**Value**
Number of active nodes	4
Total transmission time	76 s
Number of packets per second	25
Total number of packets transmitted	1900
Number of packets received directly	1884
Number of packets recovered by Node 1	7
Number of packets recovered by Node 2	1
Number of packets recovered by Node 3	3
Number of packets recovered by Node 4	5
Total number of packets recovered through forwarding	16
Total number of lost packets	0

**Table 2.** Current consumption of the wireless node.

**Component**	**Power Mode**	**Current Consumption**
Microcontroller	Idle	2.5 mA
	Sleep	0.1 μA
	Normal	4 mA

Transceiver	Idle	1.8 mA
	Sleep	0.3 μA
	Transmit, (P_out_ = +4 dBm)	25 mA
	Receive	19 mA

Memory	Stand by	25 μA
	Deep Power-down	5 μA
	Read/Write	12 mA

Accelerometer	Power on	300 μA

**Table 3.** Median received power and packet loss for the on-body node-to-node links. Node numbers correspond to the following locations: 1, front; 2, back; 3, left; 4, right.

**TX Node**	**RX Node**	**Median *P****_RX_*	**Packet Loss (%)**	**Max # Subsequently Lost Packets**
1	2	−59	0.43541	1
1	3	−55	3.4833	1
1	4	−48	4.4993	3
2	1	−60	3.7736	1
2	3	−45	3.701	2
2	4	−54	3.7736	1
3	1	−50	3.3382	1
3	2	−47	0.21771	1
3	4	−61	3.701	2
4	1	−47.5	3.5559	1
4	2	−44	0.072569	1
4	3	−58	3.4833	1

**Table 4.** Median received power and packet loss for the person-to-person links.

**TX Person**	**RX Person**	**Median *P****_RX_*	**Packet Loss (%)**	**Max # Subsequently Lost Packets**
1	2	−51	0.55866	1
1	3	−54	0.8126	1
2	1	−52	0.76181	1
2	3	−54	0.8126	1
3	1	−51	0.71102	1
3	2	−51	0.25394	1
